# Endoscopic intact removal of medium-size- or multiple bladder stones with the use of transvesical laparoendoscopic single-site surgery

**DOI:** 10.1007/s00345-018-2358-8

**Published:** 2018-06-28

**Authors:** Marek Roslan, Maciej Przudzik, Michał Borowik

**Affiliations:** Department of Urology, Faculty of Medicine, University of Warmia and Mazury, Olsztyn, Poland

**Keywords:** Urolithiasis, Bladder calculi, Endoscopy, Laparoendoscopic single-site surgery

## Abstract

**Objectives:**

To determine the feasibility and safety of performing transvesical laparoendoscopic single-site surgery (T-LESS) in patients with medium-size, hard stones or multiple stones with high burden.

**Methods:**

In this case series study, 12 patients (11 males and one female) with a mean age of 66.8 years were operated on from February 2016 to May 2017 due to bladder calculi, using the T-LESS approach with a single-port device (Tri-Port + , Olympus, Germany). Indications for this procedure were hard, medium-size, solitary stones after previous unsuccessful endoscopic lithotripsy or the presence of multiple high-burden stones. In two patients, additional procedures (diverticulectomy or a ureterocele incision) were performed simultaneously.

**Results:**

All stones were removed intact. No serious complications were observed. The mean operative time was 46 min and the postoperative hospital stay was 22 h. The mean diameter of the largest stone and the mean stone volume of each case were 24 mm and 11 cm^3^, respectively. At the mean follow-up time of 15 months, there was significant improvement of the symptoms.

**Conclusions:**

The T-LESS technique is an efficient, safe and minimally invasive procedure for intact bladder stone removal in selected patients. The method avoids the risk of urethral injury. Nevertheless, further investigation is needed to assess the wider applicability of the procedure.

## Introduction

The worldwide incidence of urolithiasis is assessed at 14%, and the number of medical interventions for this disease has increased during the last two decades. Therefore, the treatment of stone disease has become a challenge to healthcare organizations [1, 2]. In developed countries, bladder stones constitute 5% of urolithiasis [1, 3]. Cystolithiasis triggers gradually increasing lower urinary tract symptoms, and, when untreated, may lead to serious complications, including urosepsis or kidney failure. The necessity for the removal of bladder calculi is beyond doubt. There are numerous index procedures for cystolithiasis, either transurethral or percutaneous; but, open surgery remains the only way to remove the majority of larger stones intact, although it causes the most morbidity. Less morbid endoscopic treatment modalities are afflicted by the potential for incomplete stone clearance [4, 5]. The use of shockwave lithotripsy (SWL) provides the minimal morbidity, with up to 90% effectiveness; but, in one-third of the patients, additional procedures are required [1, 6]. In everyday practice, various devices are used to destroy and remove calculi. The most common are those that apply laser, ultrasonic, pneumatic and mechanical sources of energy. The introduction of the pulsed dye laser by Grasso and Bagley in 1991, followed by the introduction of the holmium laser have revolutionized the process of stone fragmentation, and have made lithotripsy procedures easier, faster and more efficient [5, 7]. Although laser lithotripsy is generally considered to be safe, bladder perforation and even serious intestinal complications may occur [8, 9]. Therefore, other solutions should be considered when decreased efficacy of standard methods or significantly prolonged operation time are expected, particularly for patients with multiple or hard calculi, creating a larger stone burden. One of the novel approaches is transvesical laparoendoscopic single-site surgery (T-LESS) that was initially introduced by Ingber et al. [10] for the removal of surgical materials eroded into the bladder. This access, using the fabric kit TriPort + , was applied successfully for the removal of unusual foreign bodies [11], and, therefore, seemed to be an attractive alternative to treat inconvenient bladder stones as well. To the best of our knowledge, this is the first series of patients treated with the T-LESS technique for the removal of specific bladder stones intact. The primary goal of this study was to determine the efficacy and safety of the method.

## Materials and methods

From February 2016 to November 2017, 75 patients (68 males and 7 females), with bladder stones were referred to our tertiary center, since we disposed the armamentarium for mechanical, ultrasound and pneumatic lithotripsy, as well as for laser disintegration, through either transurethral or percutaneous access. All the patients were diagnosed according to causative factors for cystolithiasis: The presence of bladder outlet obstruction (BOO), foreign bodies, congenital malformations or infection. Patients were examined with abdominal radiography (KUB X-ray), intravenous pyelography, or computed tomography. The diagnosis was confirmed in all patients with abdominal ultrasound and, rarely, with cystoscopy. Standard laboratory and microbiology examinations were performed.

The T-LESS approach was used in 12 patients of the group of 75 (11 males and 1 female), and calculi were removed intact.

The indications for this method were solitary hard stones (approximately 25–35 mm in diameter) after previous unsuccessful conventional endoscopic treatment, or multiple (more than 10) calculi of 15 mm mean diameter. In two patients, concomitant bladder diverticula or a large ureterocele with a stone were additional indications. Patients presented significant irritative symptoms and the majority developed bladder outlet obstruction. The bladder volume was assessed as being greater than 300 mL, to ensure that the single-port could be established properly in the bladder.

The data on operative time, hospital stay, blood loss, catheterization time, and pain measurement (the visual analog scale, VAS) were also collected (Table 1).

**Table 1:** Demographic data and outcomes

Patient no	Age (year)	BMI (kg/m^2^)	Number of stones	Diameter of the largest stone (mm)	Operative time (min)	Blood loss (mL)	Complications	Postoperative stone volume (mL)	Postoperative hospital stay (h)	Postoperative pain in VAS, day 0	Postoperative bladder catheterization (days)	Follow-up time (months)	Post void residual at last follow-up (mL)	Stone recurrence at the last follow-up visit
12	66.9 (range 39–82)	30.0 (range 26.4–38.1)	8.9 (range 1–33)	23.6 (range 16–33)	46.5 (range 15–185)	Minimal	Urethral bleeding in 1 pt.	11 (range 5–18)	22.0 (range 7–49)	1.4 (range 0–2)	6.2 (range 5–9)	14.8 (range 6–20)	20 (range 0–110)	None or small sand in 2 pts

The procedures were performed after obtaining a thoroughly designed informed consent and previous approval by the ethics committee.

### Operation technique

**Fig. 1:**
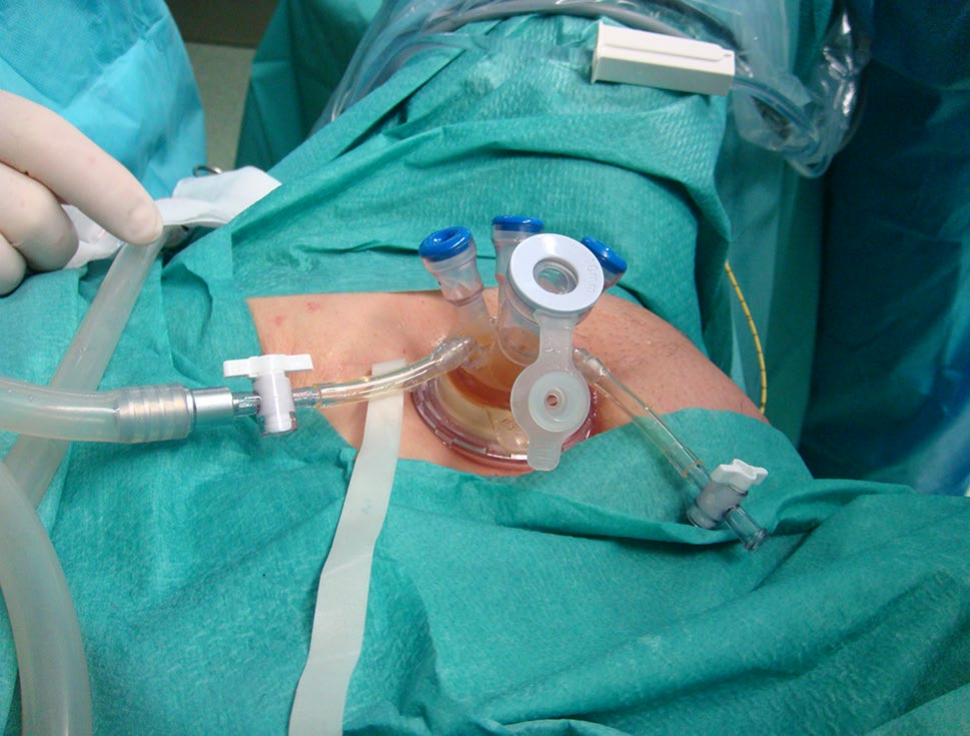
The TriPort + device fixed to the abdominal wall

Patients were operated on in the lithotomy position and under general anesthesia. A 1.5–2.5 cm skin incision was made 2–5 cm above the pubic symphysis. In all cases, an additional 1 cm rectus sheath incision was made. In a few patients, stay sutures were placed to facilitate the introduction of the single-port device (Tri-Port + , Olympus, Hamburg, Germany). Standard cystoscopy (19F cystoscope, R. Wolf, Knittlingen, Germany, with a 30° or 70° optical lens) was performed, and the introducer with the inner ring of the port was inserted through the incision directly into the bladder, under visual control. The rings of the TriPort + were connected and fixed to the abdominal wall (Fig. 1). The saline was sucked away from the bladder and a pneumovesicum was established with carbon dioxide up to a pressure of 14 mm Hg. The anatomical structures of the bladder were identified. In a patient with a diverticulum of 5 cm in diameter, an ipsilateral ureter was catheterized. In all procedures, a 10-mm 0-degree videolaparoscope, and standard, rigid laparoscopic instruments were used. In a male patient in whom diverticulectomy was also performed, an additional grasper was introduced blindly via the urethra to facilitate this step of the procedure.

**Fig. 2:**
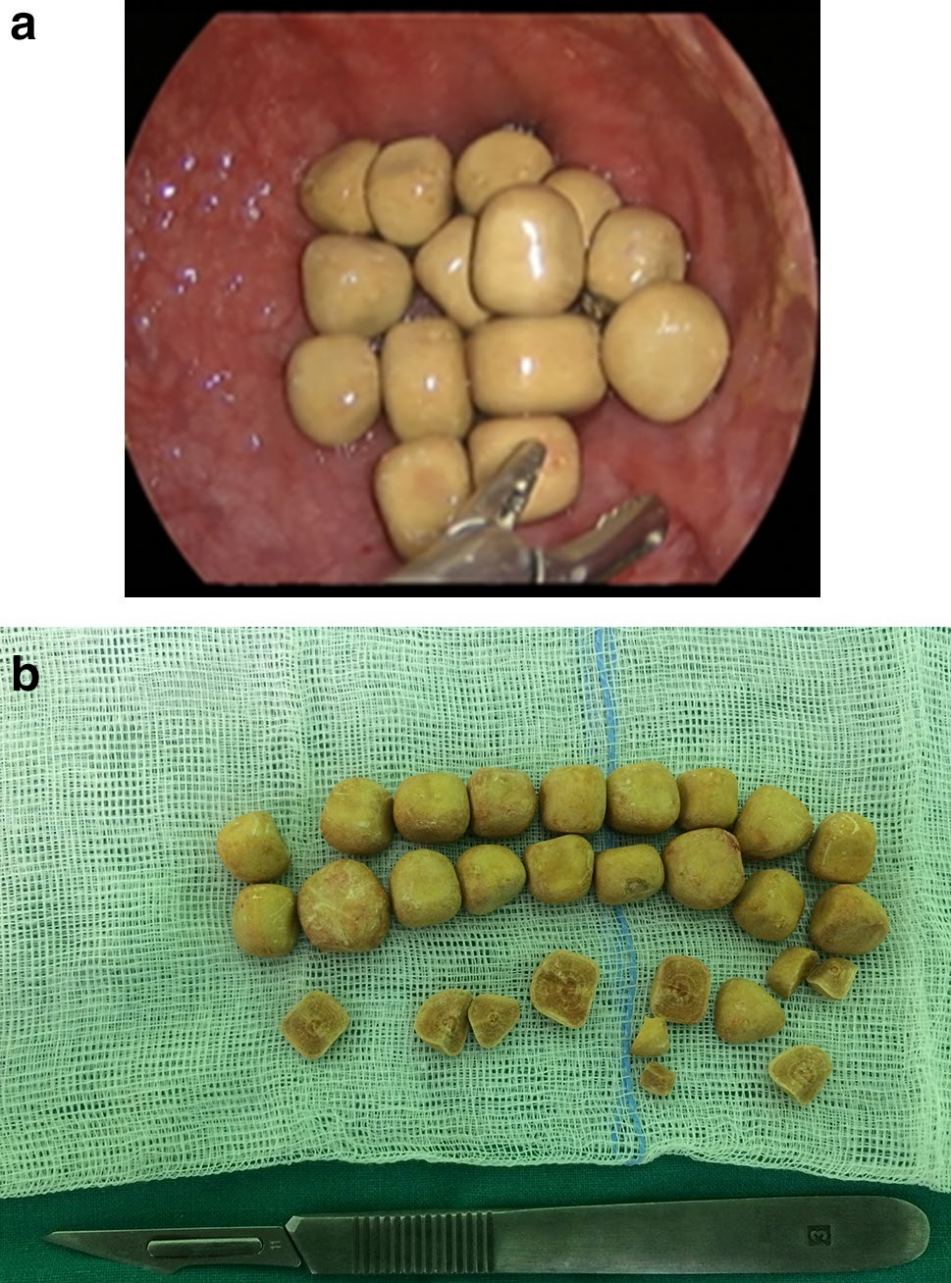
Exemplary calculi removed intact from the bladder. **a** Inside the bladder; **b** after removal

The stones were removed intact with the grasper through the TriPort + , after disconnecting the rings of the port (Fig. 2a, b). To remove small fine-grained calculi or sand, either laparoscopic suction or a bag adapted from a latex condom was used. In patients with the largest stones, it was necessary to extend the skin and rectus sheath incisions up to 3 cm. In the case when an auxiliary diverticulectomy was applied, the procedure was performed in a manner described previously by the author and co-workers [12]. In a female patient, a transverse incision of the ureterocele was made, the stone was pulled out and then extracted through the TriPort + .

After removal of all the calculi, the bladder was inspected, the pneumovesicum was evacuated, and the TriPort + was taken out. The rectus sheath and skin incision were sutured with one and two stitches, respectively. There was no need for separate closure of the bladder dome. An 18F Foley catheter was inserted to the bladder for 5–7 days. In one patient, a cystotomy tube was left for 2 days because of urethral bleeding. A course of antibiotics (quinolones or cephalosporins) was administered postoperatively, based on the results of urine culture when possible.

The 10-point visual analog pain score (VAS; 0: no pain, 10: worst possible pain) was used to assess the pain level 8 h after the operation. Follow-ups were scheduled for 4 weeks, and 3, 6 and 12 months following the operation. The evaluation included ultrasound, microbiology and uroflowmetry assessment, when necessary.

## Results

In all twelve patients, the T-LESS procedures were completed with success. No residual stone was left and no extra port was added. Table 1 presents baseline characteristics and perioperative data on patients in this group.

The operative time ranged from 13 to 185 min, and the average postoperative hospital stay was 22 h (range 7–49 h). No significant postoperative pain was noted, and the need for the administration of analgesics was minimal. Two patients were discharged on the day of operation. Blood loss was minimal in all cases except for one patient who presented a one-day bleeding from the urethra that was injured during cystoscopy. No other intra- or postoperative complications were observed. The mean diameter of the largest stone was 24 mm (16–33 mm), and the mean stone burden of each case was 11 cm^3^ (5–18 cm^3^). All the patients accepted oral food on the day of operation or the 1st postoperative day.

At the average 15-month follow-up, all patients but one presented no or insignificant post-void residual volume. In two male patients, small, fine-grained recurrent calculi were found after 12 months. Seven patients are continuing treatment with alpha-blockers. One male patient refused the TURP procedure.

## Discussion

Currently, there is no clear consensus about the optimal management of cystolithiasis. A broad spectrum of methods has been used for the treatment of bladder calculi, including open surgery, shock wave lithotripsy (SWL), and transurethral or percutaneous cystolithotripsy (cystolitholapaxy). Several factors such as stone size and number, patients’ history of treatment and general health status, availability and cost of the proper armamentarium, and, certainly, the surgeon’s preference should be considered in making the appropriate therapeutic decision. Transurethral access is the most common way to disintegrate and remove calculi from the bladder. This route often requires multiple insertions of a cystoscope that has the risk of urethral stricture, or a preceding internal urethrotomy or bladder neck incision when the obstruction is recognized.

In the present study, an innovative method of transvesical one-port intact removal of specific bladder stones is described. The drawbacks of the technique are a need of general anesthesia and the use of the suprapubic percutaneous route. However, this minimally invasive approach may have some advantages over conventional ones, particularly in the reduction of urethral traumatization or the decrease of the rate of stone recurrence.

One of the issues discussed in the literature is the need for auxiliary transurethral procedures preceding cystolithotripsy. In the work of Ener et al., 10 out of 43 patients needed urethrotomy before cystolitothripsy [13]. In addition, Ali et al. had to make a bladder neck incision in 6 of 53 patients [14]. These maneuvers lengthened the operation time. In our series, such procedures were not needed, because we performed cystoscopy with a thin 19F instrument.

Ener et al., Ali et al. and Jia et al., in whose studies of transurethral cystolithotripsy for stones of mean diameter of 25–48 mm, the reported operative times were 68, 83 and 26 min, respectively [13–15]. In our study, the mean diameter of the largest stone was 23.6 mm; however, in most patients there were multiple calculi. The postoperatively measured total stone volume for each patient was 11 mL. This volume is precise, because it was assessed as the volume of water that was replaced when the stones were placed in a calibrated dish of water. This observation suggests that the stone burden in our study is comparable to that reported by the above-mentioned authors.

In the cases presented here, the operative time was 46.5 min; but this value was increased significantly by the time taken for auxiliary procedures (diverticulectomy and ureterocele incision) in two patients. If these additional interventions would not have been performed, the mean operative time would have been 32 min. Therefore, the operative time using our technique is comparable to or even shorter than that reported by other authors.

In general, either transurethral or percutaneous endoscopic retrieval of vesical stones is associated with high efficacy, minimal invasiveness and short hospital stay, with only a minor dependence on a source of energy used for stone fragmentation.

The Holmium laser is used commonly to disintegrate urinary stones [16]. Excellent results of outpatient transurethral cystolithotripsy of large bladder stones was presented by Karami et al. in 2016. In their study, the mean time of hospital admission of patients was 6.5 h [4]. In the study by Kara et al. describing transurethral cystolithotripsy with a Holmium laser under local anesthesia, the mean hospital stay was 2.3 days [17].

In our group, the average hospital stay after the operation was 22 h, which is comparable to the results achieved when other minimally invasive techniques are used.

One of the commonly discussed aspects concerning cystolithotripsy is the risk of urethral injury, which is caused mostly by passing the cystoscope with a stone fragment through the urethra [14]. In our technique, the widest instrument inserted via the urethra is the 19F cystoscope, and this maneuver is performed only once at the beginning of the procedure. The other action made through the urethra is the placement of the 18Fr Foley catheter. Therefore, the risk of urethral injury is decreased significantly. We should add that in a male patient with the bladder diverticulum we inserted transurethrally a 5-mm-in-diameter laparoscopic grasper. Since 2011, we have performed several T-LESS diverticulectomies, and we ensured that the delicate passing of the grasper through the urethra is a fast and safe maneuver, even in enlarged prostate glands.

Although lasers are considered as safe and efficacious tools to fragment calculi, they are also not free of complications. Althunayan and colleagues reported a case of bladder perforation that resulted in urinary diversion [8]. Vaidyanathan et al. described a tetraplegic patient who underwent laser cystolithotripsy of a 15 mm stone, and then developed life-threatening complications requiring several endoscopic and abdominal surgeries, and a 21-day stay in the intensive care unit [9]. To avoid these disadvantages, some authors have used the percutaneous suprapubic approach. PCCL with the Amplatz sheath introduced directly to the bladder is an established technique [13, 16].

Percutaneous cystolithotomy to extract the entire stone burden using a laparoscopic entrapment bag was accomplished by Miller and Park in four patients with augmented bladders. Conversion to open surgery was required in one patient because of tearing of the entrapment sac [18]. An interesting suprapubic access for the removal of bladder stones in 25 patients was described by Tan et al. in 2014. The authors inserted percutaneously the 30F Amplatz sheath, introduced a laparoscopic entrapment bag into the bladder, and stones were placed into the bag with the flexible cystoscope that was installed transurethrally. Calculi were crushed in the bag and they were removed from the bladder with the bag. Although the average operative time was 102 min, this maneuver prevented stone remnants to be left in the bladder. No significant complications were observed, and no stone recurrence was noticed during the mean follow-up of 22 months [19].

These data may suggest that removal of the calculi intact potentially decreases the recurrence rate of the disease. Therefore, the percutaneous transvesical laparoendoscopic single-port approach seemed to be an attractive alternative in such patients.

With reference to patients treated in our study, those who underwent additional procedures at the same session may need to be taken into particular consideration. In a patient with a bladder diverticulum of 5 cm in diameter, over 30 calculi up to 16 mm in diameter were diagnosed. During the operation, all the smaller stones were removed first via the TriPort + , and the remaining concrements greater than 1 cm were removed after diverticulectomy. At the 19-month follow-up, no bladder diverticulum or stone were found. In a female patient, in whom a transverse incision of the right ureterocele was carried out to pull out a stone of 30 mm in diameter, neither vesicoureteral reflux nor infection were observed at the one-year follow-up. These two patients presented no obstructive symptoms and did not require surgical treatment of BOO.

Although cystolithiasis is considered an absolute indication for bladder neck incision or TURP [20], those patients who refuse prostatic surgery or are diagnosed with important comorbidities may be successfully treated with medical therapy after bladder stone clearance if they have no significant postvoid residual urine volume (PVR) [1]. In our center, we routinely perform bladder neck incision or TURP following cystoslithotripsy. Nevertheless, in relevant group we decided to delay a prospective bladder outlet surgery due to the combining procedures lengthens the operative time and may increase the risk of complications. Moreover, we scheduled the patients to assess the decrease of postoperative symptoms and capacity of PVR urine volume. We should mention that in the patient with neurogenic bladder a transurethral catheter was remained. Ultimately, during a follow-up only one man presented an increased PVR, but he refused surgical treatment.

There are some limitations of this work, as it reports a small number of patients, and, because of the focus of the treatment on the T-LESS procedure, there is no a comparison group. Nevertheless, the T-LESS approach remains an innovative and successful method. The study presents our initial experience with this novel technique that may be applied to the treatment of patients with bladder calculi who are unfit for standard transurethral therapy, particularly in centers that do not have at their disposal expensive laser lithotripsy. The method we have described allows the removal of medium-size, intact stones, and decreases the risk of stone recurrence over a relatively long follow-up period. The T-LESS access has the capacity to shorten the operative time without lengthening the hospital stay. Patients with some concomitant bladder pathology can be treated during the same session, utilizing a minimally invasive procedure. Moreover, by its nature, the technique avoids the risk of urethral injury, and may potentially be safer for patients with neurogenic bladders.

A potential future application of the T-LESS bladder stone retrieval may be the concomitant enucleation and removal of an enlarged prostate gland without morcellation. This procedure was first described by Desai et al. [21]. We surmise that even glands greater than 5–6 cm in diameter could be extracted intact or after halving inside the bladder. Additionally, although the procedure seems to be cheaper in comparison to laser cystolithotripsy, its real cost-effectiveness would require further investigation.

## Conclusions

The T-LESS procedure for bladder stone removal is a safe, effective, reproducible and minimally invasive procedure. It allows the achievement of complete stone clearance without leaving stone remnants that could have served as nuclei for new stone formation. The method may reduce the operative time in patients with hard stones, and represents a potential advantage over other techniques in cases with multiple, medium-size calculi. Moreover, the method provides the opportunity to perform simultaneously additional procedures such as diverticulectomy or ureterocele incision. Nevertheless, further studies are needed to assess the broader applicability of the method.
